# *Bacillus megaterium* and diatom improve mineral mining area soil quality and root biomass individually but show slightly inferior combined effects

**DOI:** 10.3389/fpls.2025.1716914

**Published:** 2025-12-16

**Authors:** Yuyang Li, Jiachen Pan, Xinran Shi, Changxin Tang, Xuejia Zheng, Chen Li, Jinhua Zhao, Zhi Dong, Qicong Wu, Congzhi Zhang

**Affiliations:** 1 Co-Innovation Center for Soil-Water and Forest-Grass Ecological Conservation in Yellow River Basin of Shandong Higher Education Institutions, College of Forestry, Shandong Agricultural University, Tai’an, China; 2 College of Soil and Water Conservation, Beijing Forestry University, Beijing, China; 3 Government of Lucun Town, Yiyuan County, Zibo, China; 4 Ecological Environment Geo-Service Center of Henan Geological Bureau, Zhengzhou, China; 5 Key Laboratory of Crop Water Physiology and Drought-Tolerance Germplasm Improvement, Ministry of Agriculture and Rural Affairs, College of Agronomy, Shandong Agricultural University, Tai’an, China; 6 State Experimental Station of Agro-ecosystem in Fengqiu, State Key Laboratory of Soil and Sustainable Agriculture, Institute of Soil Science, Chinese Academy of Sciences, Nanjing, China

**Keywords:** biological soil crusts, mine restoration, soil quality index, plant biomass, soil nutrients

## Abstract

Biological soil crusts (biocrusts) promote plant growth by improving soil structure and regulating soil nutrients. However, their effects on soil quality in mining subsidence areas remain poorly understood. The present study aimed to address this research gap by comparing the differences in soil nutrients, enzyme activities, soil quality, and plant growth following treatment with different types of biocrusts (control, diatom, *Bacillus megaterium*, and diatom-*B. megaterium* biocrusts) in mining subsidence areas. The results indicated that, compared to control biocrust treatment, individual treatment with diatom biocrust and *B. megaterium* biocrust significantly enhanced the organic carbon content, total nitrogen content, and invertase activity in the crust layer and increased the soil quality index values to 0.52 and 0.54, respectively. Diatom biocrust treatment was associated with a significant increase in ryegrass root biomass, which be linked to its improvement of soil structure. Ryegrass root biomass increased to 22.69 g, this effect was mediated by secretion of extracellular polymeric substances from the biocrust. In contrast, diatom-*B. megaterium* biocrust treatment increased crust roughness by 36.6% and improved soil moisture content by 18.7%, thereby further enhancing ryegrass root biomass. However, the diatom-*B. megaterium* biocrust treatment did not improve the soil quality index, despite increasing root biomass. Overall, these findings highlight the importance of applying biocrusts for soil remediation and plant growth in mining subsidence areas, providing a scientific basis for sustainable management and ecological restoration of degraded soils.

## 1 Introduction

Mining subsidence areas refer to land surfaces undergoing sinking, fracturing, or collapse due to the displacement and deformation of overlying rock layers caused by subsurface resource extraction (Wang et al., 2019). This process substantially damages pre-existing vegetation and soil ecosystems, thereby enhancing erosion and ecological deterioration (Guo et al., 2019; Wang et al., 2013). Specifically, mineral extraction harms plant life by disrupting root networks and modifying ground cover. Concurrently, it degrades soil by disturbing its structure, reducing microbial diversity, and accelerating nutrient depletion. Biological soil crusts (BSCs), which are intricate soil surface communities comprising a network of microorganisms (such as algae, lichens, and mosses) and soil particles, are estimated to cover approximately 12% of the Earth’s land area (Chamizo et al., 2012). These crusts are vital for stabilizing soil, enhancing fertility, and regulating microhabitat conditions in the topsoil layer. Although BSCs are known for soil enhancement capabilities, their utilization in ecological rehabilitation projects remains constrained. A primary limitation is the inadequate comprehension of how they interact synergistically with vegetation to improve soil quality.

Different types of BSCs exert varying effects on soil properties and plant development, which could be attributed to differences in microbial physiology and metabolism. For example, bacterial crusts improve soil aggregation. This occurs through hydrophobic proteins (hydrophobins) secreted by bacterial filaments, which bind soil particles together to form larger aggregates (Rillig et al., 2014). These crusts also affect nutrient cycles by expediting litter degradation by ligninolytic enzymes (e.g., laccase) and sequestering carbon through extensive mycelial networks (Kögel-Knabner, 2017). Ectomycorrhizal bacterial in these crusts additionally support plant nitrogen uptake through symbiotic relationships (Van Der Heijden et al., 2015). Conversely, algae-dominated crusts promote soil stabilization through the secretion of polysaccharides that bind particles. They also strengthen seed retention and sprouting, facilitating plant re-establishment (Chamizo et al., 2018). Moreover, algal crusts convert atmospheric nitrogen using nitrogenase and increase soil organic carbon content through photosynthetic activity, thereby enhancing soil nutrient status (Elbert et al., 2012).

Current investigations on the ecological roles of BSCs in soil systems are primarily limited to arid and semi-arid environments, with insufficient research conducted in mining-induced subsidence areas. These crusts commonly colonize the interstitial spaces between plants and the soil surface (Belnap et al., 2001), where they modify edaphic characteristics to generate supportive conditions for seedling establishment and subsequent vegetation development (Fick et al., 2020). BSCs also modulate topsoil microenvironments, protect young plants from burial by windblown sand, and influence the structure of soil microbial assemblages. Previous research suggests that although BSCs might not substantially increase overall plant species diversity, they can restructure the composition of the herbaceous community and facilitate beneficial successional processes (Bowker, 2007).

Perennial ryegrass (*Lolium perenne* L.), a cool-season grass with an extensive root network, prevents soil compaction by enhancing aeration and hydraulic conductivity (Bodner et al., 2014). It also diminishes surface crack propagation and erosion susceptibility in subsided terrain (Huang et al., 2003; Petersen et al., 1970). These beneficial characteristics establish ryegrass as a valuable restoration species for degraded mining landscapes. Prior research indicates that *Bacillus megaterium* crusts can mitigate soil nutrient deficiencies and stimulate ryegrass germination and development. Nevertheless, thus far, only a few studies have assessed the influence of BSCs on the soil quality index (SQI) of mining subsidence areas, particularly regarding the multifaceted impacts of BSCs on soil health.

Hence, in the present study, a pot-based experiment was conducted to assess how Bacterial crusts, algal crusts, and mixed biological crusts influence SQI and ryegrass growth performance in soils from mining subsidence areas. The study aimed to: (1) determine the comparative efficacy of distinct BSC types in enhancing SQI of these affected areas and (2) investigate the relationships between BSC-induced changes in soil properties and ryegrass growth. Four experimental treatments were implemented: an untreated control, treatment with a diatom crust, treatment with a *B. megaterium* crust, and treatment with a combined diatom-*B. megaterium* crust. Soil physical structure, nutrient status, enzymatic activity profiles, and various ryegrass growth metrics were evaluated. The results are expected to provide a theoretical foundation and practical strategies for ecological rehabilitation in mining subsidence-impacted regions.

## 2 Materials and methods

### 2.1 Overview of the study area

Soil for testing was collected in September 2022 from the Nanyang Lake Farm (35°19′ N, 116°37′ E), Rencheng District, Jining City, Shandong Province, a typical coal mining subsidence area. The soil type was classified as Typic Hapludalfs (USDA Soil Taxonomy). This area is a typical coal mine subsidence zone with poor soil structure and low aggregate stability. The region experiences a warm, temperate, semi-humid, continental monsoon climate, with a mean annual temperature of 13.5 °C and a mean annual precipitation of 670 mm, 65% of which occurs from June to August. Soil samples (0–20 cm depth) were collected using a five-point sampling method, with at least 2 m distance between adjacent sampling points. The initial soil moisture content (SWC) was 12.82%, pH 7.03, SOC 13.89 g/kg, soil total nitrogen (TN) content was 1.14 g/kg, soil total phosphorus (TP) content was 110.38 mg/kg, soil bulk density (BD) was 1.35 g/cm³.

Pot experiments were conducted from March to May 2023 at the Comprehensive Experimental Base of Shandong Agricultural University, Tai’an City, Shandong Province (35°38′–36°28′ N, 116°02′–117°59′ E). The experimental site has a warm, temperate, semi-humid, continental monsoon climate, with a mean annual sunshine duration of 2627.1 h (58% sunshine percentage), mean monthly temperature of 16.0 °C, and mean monthly precipitation of 44.2 mm during the growth period. To eliminate the interference of natural factors (such as rainfall and temperature fluctuations) on the experimental soil and plants, the experiments were conducted in a climate-controlled greenhouse.

### 2.2 Experimental materials and experimental design

A pot experiment was conducted with *L. perenne* cv. Winter Grazing 70 and four treatment types: control (CK), diatom crust (DI), *B. megaterium* crust (BA), and mixed diatom-*B. megaterium* crust (DB), with triplicate pots per treatment. Twelve rectangular plastic containers (40 cm × 20 cm × 20 cm; 16,000 cm³ volume) were prepared. Air-dried soil was sieved (2 mm mesh) to remove debris, homogenized, and portioned. A total of 21.6 kg of the prepared soil was added to each pot to achieve field-representative bulk density (1.35 g cm^-3^). The treatments were administered as 2.5 L surface sprays as follows: CK: deionized water only; DI: 8 g diatoms in an aqueous suspension; BA: 8 g *B. megaterium* in an aqueous suspension; DB: a mixed aqueous suspension of 4 g diatoms + 4 g *B. megaterium*. The inoculation dosage of 8 g/pot was determined based on preliminary experiments conducted by our research group (Zheng et al., 2024). This dosage was found to form a continuous and visible crust on the soil surface without adversely affecting seedling germination. Ryegrass seeds were surface spread at 20.0 g per pot (equivalent to 25 g m^-2^). Irrigation was performed daily (days 1–3), followed by tri-weekly watering to maintain soil moisture at 60–70% of field capacity.

### 2.3 Sample collection

After 40 days of growth, ryegrass, biological crust (biocrust), and soil samples were collected. A 10 cm × 10 cm sampling area was designated in each pot. By using a sampling shovel, the biocrust (i.e., the surface layer containing the separable crust material) was carefully peeled along the edges and then removed with a small spade. A soft brush was used to gently dislodge the adhered soil particles beneath the crust, ensuring that the soil particles naturally detached to yield intact crust samples (Huang et al., 2023). Three replicates were sampled per treatment, and a total of 12 crust samples were obtained. Below the crust, soil samples were collected to a depth of 10 cm (from the crust-soil interface). Three replicates per treatment were taken, resulting in 12 soil samples. These samples were divided into two subsamples: one subsample was air-dried in the shade (with partial sieving through a 2 mm mesh) for measuring physicochemical properties, and the other subsample was utilized as a fresh sample for soil aggregate analysis. At the harvesting time (40 days), ryegrass reached the mature stage. For each treatment, three plants with uniform growth were randomly selected. The aboveground and underground tissues were separated using a sterile scalpel. Roots were carefully rinsed with deionized water, dried at 65 °C to constant weight, and weighed to determine biomass.

### 2.4 Soil property determination

#### 2.4.1 Determination of physical and chemical indices of soil

The soil saturated water content (SWC) was measured using the ring knife immersion method. Soil mechanical aggregates were analyzed by dry sieving, and water-stable aggregates were determined through wet sieving. Both sieving methods yielded four particle size fractions: >2 mm, 2–0.25 mm, 0.25–0.053 mm, and<0.053 mm. The mass percentage of aggregates in each size category was quantified. Three key stability indices; i.e., mean weight diameter (MWD), geometric mean diameter (GMD), and percentage of aggregate destruction (PAD), were subsequently calculated according to the Equations 1–3 (Wang et al., 2024):

(1) $$MWD=\sum_{i=1}^{n}\left(X_{i}\times W_{i}\right)$$

(2) $$GMD=\exp\left(\sum_{i=1}^{n}W_{i}\ln X_{i}\right)$$

where n represents the number of particle size groups, *X_i_* denotes the average diameter of each aggregate fraction (mm), and *W_i_* is the percentage of aggregates in that fraction.

(3) $$PAD=\left(DR_{0.25}-WR_{0.25}\right)/DR_{0.25}\times 100\%$$

where DR_0.25_ refers to the content of mechanically stable aggregates >0.25 mm (%) and WR_0.25_ represents the content of water-stable aggregates >0.25 mm (%).

Soil samples were air-dried, ground, and sieved prior to analysis. Soil pH was measured in a 2.5:1 water-to-soil suspension. Soil organic carbon (SOC) content was determined using the potassium dichromate (K_2_Cr_2_O_7_) external heating method (Nelson and Sommers, 1983). Total nitrogen (TN) content was quantified by the Kjeldahl digestion method. Total phosphorus (TP) content was analyzed using the sodium hydroxide fusion–molybdenum antimony anti-colorimetric method (Pang et al., 2003). Alkaline nitrogen (AN) content was measured by NaOH alkaline hydrolysis, and available phosphorus (AP) content was estimated using the molybdenum antimony colorimetric method.

#### 2.4.2 Determination of soil enzyme activity

Purchase kits from Suzhou Grace Biologics Co., Ltd., China, and follow the instructions provided in the kit to determine the activity of β-soil sucrase (SC) and urease (UE). Referring to the manufacturer’s instructions and the research method of Bell et al. (2013), SC activity is assessed by detecting the amount of reducing sugars produced by the reaction, briefly, 5.00 g of fresh soil with 15 ml of 1% CMC solution incubated in 0.1 M acetic acid buffer (pH 5.5) for 24 h at 50 °C. After incubation, the mixture is centrifuged and filtered. Take 1 ml of filtrate and react with 2 ml of DNS reagent in a boiling water bath for 10 minutes. After cooling, absorbance was measured at 540 nm. The concentration of reducing sugars is quantified by glucose standard curves. Enzyme activity is expressed as the number of milligrams of glucose released per 24 hours per gram of dry soil (mg glucose/g dry soil, 24 hours^-1^). UE activity was assessed by detecting ammonium ions produced by the reaction as follows: 5.00 g of fresh soil was incubated with 5 ml of 10% urea solution and 10 ml of citrate buffer (pH 6.7) for 24 hours at 37 °C. Subsequently, 25 ml of 1 M KCl solution was added to terminate the reaction and ammonium was extracted. The soil suspension is shaken for 1 hour, then filtered. Take 1 ml of filtrate and mix it with 5 ml of phenol-nitrososodium ferrocyanide solution and 5 ml of alkaline hypochlorite. The mixture is kept warm at 37 °C for 30 minutes to promote color formation. The absorbance of the blue complex was measured at a wavelength of 630 nm. The content of ammonium nitrogen was determined using the (NH_4_) _2_SO_4_ standard curve. Activity is expressed in milligrams of ammonium nitrogen per gram of dry soil per 24 hours (mg NH_4_^+^ -N g^-1^ dry soil 24 h^-1^).

### 2.5 Characteristic indices of the biocrust

The hardness of the biocrust was measured using a hand-held soil penetrometer (Model 06.03, Eijkelkamp, Giesbeek, the Netherlands). Surface roughness of the soil crust (CR) was assessed through the chain method (Ali, 1993), which involves placing a chain with an original length L1 (mm) on the crust surface. The chain deforms because of surface roughness, and its horizontal projected length L2 (mm) decreases with increasing surface roughness. The surface roughness index Cr (%) calculated according to Equation 4:

(4) $$Cr=\frac{1-L_{2}}{L_{1}}\times 100$$

The chain used in this experiment had the following dimensions: L_1_: 43.0 mm, diameter: 1.1 mm, and node length: 1.0 mm. Measurements were conducted in the inter-plant spaces of ryegrass to avoid interference from plant root systems.

The basic physicochemical properties and enzyme activity indices of the biocrusts were compared with those of soil.

### 2.6 Data analysis

#### 2.6.1 Soil quality index

Soil quality was assessed based on the SQI by using the minimal dataset (MDS) approach reported by Liu et al. (2014). The procedure included four sequential phases. Initially, the MDS was established as follows: 12 soil parameters (SWC, MWD, GMD, PAD, pH, SOC, TN, TP, AN, AP, SC, and UE) were identified from the existing literature (Zhao et al., 2011) and subjected to principal component analysis (PCA). In the SQI calculation system of this study, PAD, pH is considered a negative indicator of “the smaller the better”. The components meeting the Kaiser criterion (eigenvalues ≥1) were retained (Brejda et al., 2000). In each retained component, variables exhibiting absolute loading values within 10% of the maximum loading per component were considered significant. Among the highly correlated variables (r ≥ 0.60) in a single component, the variable explaining the greatest cumulative variance was included in the MDS. This process yielded four MDS indicators: SC, GMD, SOC, and TP. Subsequently, weights (W_i_) for each MDS variable were determined by their factor communalities, calculated as the communality of each variable divided by the sum of all MDS communalities. Next, individual MDS indicators were transformed using piecewise linear scoring functions (Qi et al., 2009) to derive normalized scores (S_i_) on a scale of 0 to 1.0. Finally, the overall SQI value is calculated according to Equation 5 (Doran and Parkin, 1994):

(5) $$SQI=\sum_{i=1}^{n}Wi\times Si$$

where W_i_ is the weight of indicator i, S_i_ is the normalized score, and n is the number of MDS variables.

#### 2.6.2 Essential procedures for the Grey Relational Analysis

The intricate plant-soil interactions were assessed by Grey Relational Analysis (GRA) as follows. Grey Relational Analysis (GRA) is particularly suitable for handling the nonlinear system in this study, which involves a limited sample size and multiple indicators. It can holistically evaluate the proximity of different treatments to an ideal plant-soil system, thereby effectively complementing the limitations of SQI (which focuses solely on soil properties) and regression analysis (which emphasizes pairwise relationships). GRA involves computing grey relational grades derived from relational factors by using constrained quantitative datasets. The standard GRA protocol comprises the following six sequential stages (Wang et al., 2016).

(1) Indicator selection.

Evaluation metrics were selected according to two principles. First, the chosen indices must comprehensively represent system attributes. This investigation incorporated BSC, soil, and plant parameters, designated as *X* (k = 1,2,3…). Second, the indices should be statistically independent to prevent effect superposition.

(2) Reference sequence definition.

The reference sequence (*X*_0_) is an idealized benchmark comprising each indicator’s optimal value within the system. “Larger-the-better” indicators yield superior outcomes with higher values, while “smaller-the-better” indicators are favorable at lower values. This prioritization schema was subsequently applied.

(3) Data standardization.

The following equations were used for standardization (Kadier et al., 2015, **Supplementary Table S3**):

If the index is of the larger-the-better variety, then *X_i_*(*k*) is calculated according to Equation 6:

(6) $$X_{i}\left(k\right)=\frac{X\left(k\right)-minX_{i}\left(k\right)}{maxX_{i}\left(k\right)-minX_{i}\left(k\right)}$$

If the index is of the smaller-the-better variety, then *X_i_*(*k*) is calculated according to Equation 7:

(7) $$X_{i}\left(k\right)=\frac{maxX_{i}\left(k\right)-X\left(k\right)}{maxX_{i}\left(k\right)-minX_{i}\left(k\right)}$$

where *X_i_*(*k*) is the standardization result, *X*(*k*) is the raw data result, max*X_i_*(*k*) is the maximum value, and min*X_i_*(*k*) is the minimum value.

(4) Absolute deviation calculation.

The absolute deviation sequences was calculated using Equations 8, 9) (Fang et al., 2014):

(8) $$\Delta min=\;min_{i}min_{k}\left|X_{i}\left(k\right)-X_{0}\left(k\right)\right|$$

(9) $$\Delta max=\;max_{i}max_{k}\left|X_{0}\left(k\right)-X_{i}\left(k\right)\right|$$

where *X*_0_(*k*) is the priority sequence, *X_i_*(*k*) is the standardization results for the original data, Δ*max* is the maximum deviation, and Δ*min* is the minimum deviation.

(5) Relational grade computation.

The correlation coefficient between the priority sequence *X_0_*(*k*) and the comparative sequence *X_i_*(*k*) denoted by *η_i_*(*k*) calculated using Equation 10 (Wang et al., 2016, **Table 2**):

(10) $$\eta_{i}\left(k\right)=\frac{\;min_{i}min_{k}\left|X_{i}\left(k\right)-X_{0}\left(k\right)\right|+\rho max_{i}max_{k}\left|X_{0}\left(k\right)-X_{i}\left(k\right)\right|}{\left|X_{i}\left(k\right)-X_{0}\left(k\right)\right|+\rho max_{i}max_{k}\left|X_{0}\left(k\right)-X_{i}\left(k\right)\right|}$$

where *ρ* is the distinguishing coefficient, ∈ [0,1], and the value is considered to be 0.5 (Fang et al., 2014).

(6) Grey relational grade.

The relational grade (*r*) of each factor between the reference sequence and the comparative sequence was calculated according to the Equation 11 (Kadier et al., 2015):

(11) $$r=\frac{1}{n}\sum_{i=1}^{n}\eta_{i}$$

One-way variance was used to study the effects of each crust treatment on the crust layer and soil indicators. Statistical analyses were performed using SPSS 13.0 software (SPSS Corporation, Chicago, IL, USA), with one-way analysis of variance to test whether the differences were significant. The effects of each biocrust treatment on soil structure and nutrients were evaluated through soil indicators, plant growth, and their interactions. Data with a normal distribution were tested using the Shapiro-Wilk test, and Duncan’s multiple range test was utilized for multiple comparisons (P< 0.05). The R program was used for calculating linear regression and random forest.

## 3 Results

### 3.1 Indices of the biocrusts

**Figure 1:**
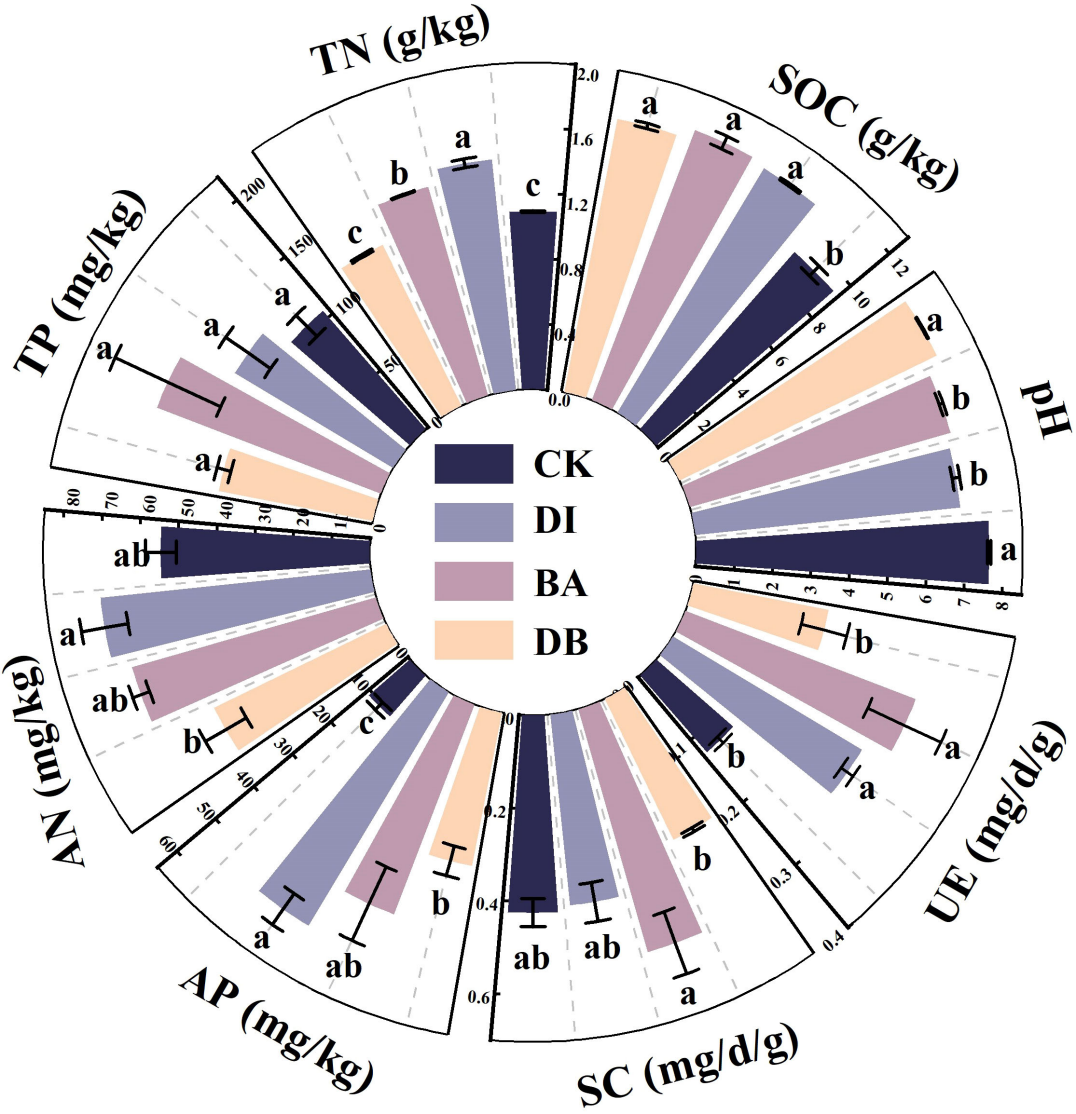
Chemical and biological indicators of the biological soil crusts. pH; SOC, soil organic carbon; TN, total nitrogen; TP, total phosphorus; AN, alkaline nitrogen; AP, available phosphorus; SC, sucrase activity; UE, urease activity. CK, without added crusting agent; DI, added diatom crusting agent; BA, added bacillus crusting agent; DB, added mixed diatom-*B. megaterium* crust. Different letters indicate significant differences at P< 0.05.

The results showed significant differences in crust physical properties and nutrient content across biocrust treatments (**Table 1**, **Figure 1**). Compared to the CK treatment, the BA treatment increased crust hardness and thickness by 15.3% and 14.6%, respectively. The DB treatment exhibited a 36.26% increase in crust roughness compared to the CK treatment (P< 0.05), which was the largest improvement among all biocrust treatments. Spraying crusting agents significantly enhanced the multiple physical indicators of the crust layer. The DB treatment showed the most pronounced increase in SWC (+18.9%) and MWD (+91.1%) compared to the CK treatment. The DI treatment achieved the highest value for MWD (12.8 mm). The DI-treated crust had a hardness of 0.39 Pa (11.4% higher than that of the CK-treated crust), while the DB treatment resulted in a 36.6% significant improvement in surface roughness over CK treatment (P = 0.003).

**Table 1:** Physical indices of the crust following treatment with different biocrusts.

Treatments	CK	DI	BA	DB
Biocrust hardness (pa)	0.35±0.02b	0.39±0.04ab	0.4±0.03a	0.37±0.04ab
CR (%)	4.45±0.97b	5.20±1.16ab	5.08±1.00ab	6.08±1.13a
Biocrust SWC (%)	11.44±0.19b	12.80±0.53ab	13.08±0.49ab	13.60±0.78a
Biocrust MWD (mm)	1.68±0.09c	2.55±0.05a	2.15±0.08b	2.14±0.11b
Biocrust GMD (mm)	0.79±0.04c	1.51±0.07a	1.20±0.05b	1.14±0.02b
Biocrust water-stable MWD (mm)	0.52±0.04b	1.01±0.13a	0.90±0.05a	0.92±0.02a
Biocrust water-stable GMD (mm)	0.28±0.06b	0.71±0.05a	0.63±0.02a	0.67±0.04a
Biocrust PAD (%)	43.24±14.82a	1.21±3.17b	8.37±1.92b	1.32±4.20b

CR, biocrust roughness; SWC, saturated water content; MWD, mean weight diameter of aggregate; GMD, geometric mean diameter of aggregate; PAD, percentage of aggregate destruction. CK, without added crusting agent; DI, added diatom crusting agent; BA, added bacillus crusting agent; DB, added mixed diatom-*B. megaterium* crust. Different letters in the same column indicate significant differences at P< 0.05.

Both DI and BA treatments significantly reduced crust pH compared to the CK treatment (P< 0.05), with the DI treatment exhibiting the greatest decrease in pH (**Figure 1**). DI and BA treatments also enhanced SOC, TN, and AP contents relative to the CK treatment. Specifically, the SOC, TN, and AP contents in the DI-treated crust were 21.4%(P<0.001) 30.4%(P<0.001) and 400.7%(P<0.01) higher than those in the CK-treated crust, respectively (**Figure 1**).

No significant differences in SC activity were observed among DI, BA, DB, and CK treatments, with the enzyme activity level in the following order: BA > CK > DI > DB (**Figure 1**). DI and BA treatments significantly increased UE activity by 16.1% and 41.6%, respectively, compared to the CK treatment (P< 0.001), with the enzyme activity in the following order: BA > DI > DB > CK (**Figure 1**).

### 3.2 Soil physical properties

The GMD, water-stable MWD, and water-stable GMD in DI, BA, and DB treatments were significantly higher than those in the CK treatment (**Table 2**). Specifically, the DI treatment exhibited 34.5% (P<0.001), 44.0% (P<0.001), and 37.0% (P<0.001) increase in the GMD, water-stable MWD, and water-stable GMD, respectively, compared to the CK treatment. Concurrently, the PAD percentage in the DI treatment was significantly elevated by 58.6% relative to that in the CK treatment (P< 0.05, **Table 2**). The DI treatment significantly increased the PAD, indicating that although the crust formed was very effective in physical covering (manifested as high MWD), the cemented substances inside it contributed limited to the stability of the water.

**Table 2:** Physical indices of the soil following treatment with different biocrusts.

Treatments	CK	DI	BA	DB
SWC (%)	14.27±0.77a	16.68±1.52a	16.87±0.97a	17.35±1.56a
MWD (mm)	1.89±0.06c	2.19±0.06bc	2.31±0.11b	2.85±0.14a
GMD (mm)	1.10±0.05c	1.48±0.09b	1.69±0.08b	2.03±0.15a
Water-stable MWD (mm)	1.34±0.14b	1.93±0.13a	2.11±0.16a	1.94±0.13a
Water-stable GMD (mm)	0.54±0.04b	0.74±0.01a	0.69±0.02a	0.66±0.02a
PAD (%)	19.60±2.13a	8.12±1.26b	16.74±0.42a	23.64±4.57a

SWC, saturated water content; MWD, mean weight diameter of aggregate; GMD, geometric mean diameter of aggregate; PAD, percentage of aggregate destruction. CK, without added crusting agent; DI, added diatom crusting agent; BA, added bacillus crusting agent; DB, added mixed diatom-*B. megaterium* crust. Different letters in the same column indicate significant differences at P< 0.05.

### 3.3 Soil chemical properties

**Figure 2:**
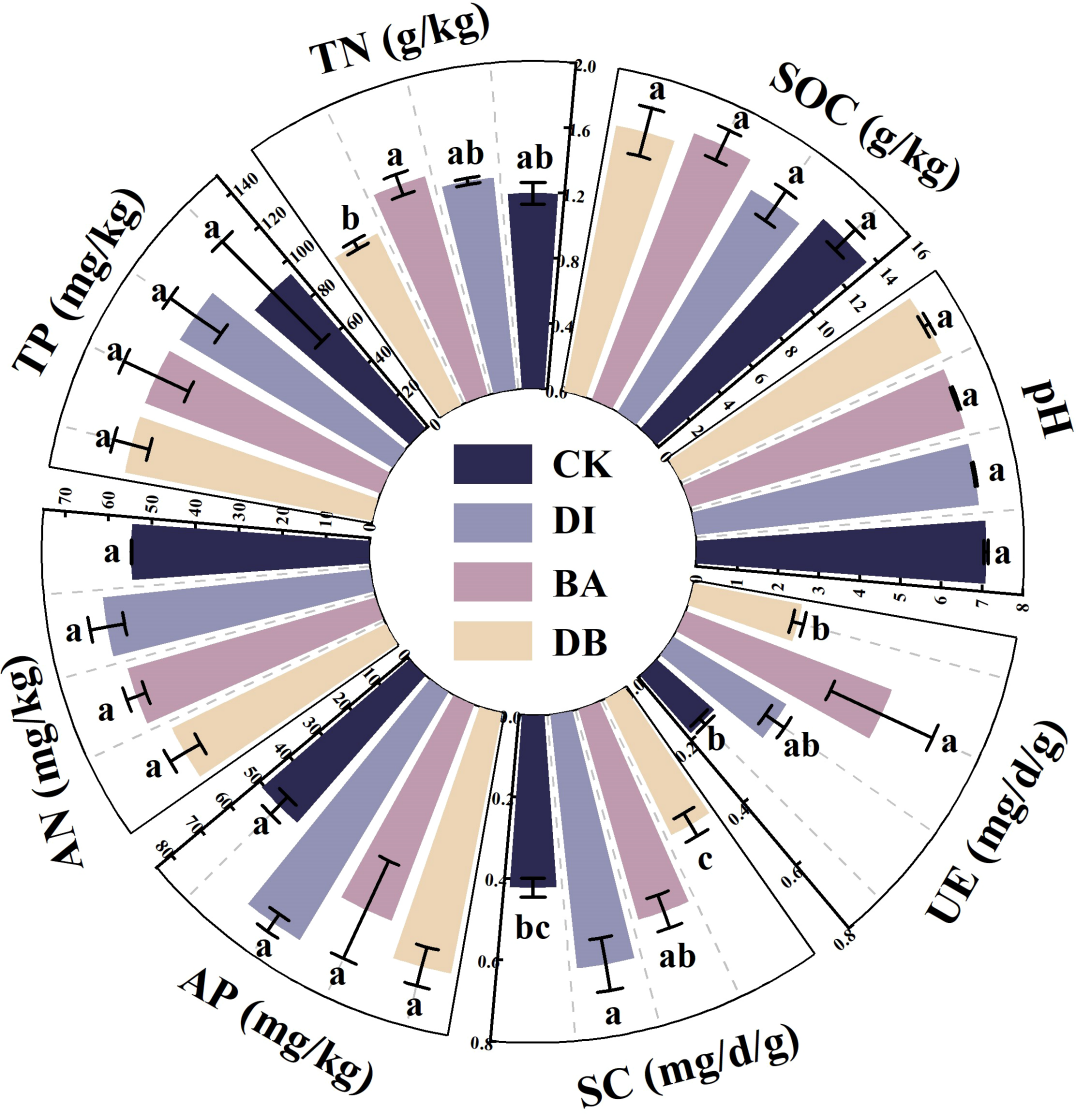
Chemical and biological indicators of the soil. pH; SOC, soil organic carbon; TN, total nitrogen; TP, total phosphorus; AN, alkaline nitrogen; AP, available phosphorus; SC, sucrase activity; UE, urease activity. CK, without added crusting agent; DI, added diatom crusting agent; BA, added bacillus crusting agent; DB, added mixed diatom-*B. megaterium* crust. Different letters indicate significant differences at P< 0.05.

The BA treatment showed a significantly higher soil TN content than the CK treatment (P< 0.05, **Figure 2**). The DI treatment exhibited the most prominent increase in AN (+12.8%) and AP (+48.0%) contents compared to the CK treatment (**Figure 2**). Both DI and BA treatments reduced soil pH relative to the CK treatment, with the DI treatment recording the lowest pH value (**Figure 2**).

### 3.4 Soil biological properties

The DI treatment showed a 47.9% increase in soil SC activity compared to the CK treatment (P< 0.05), with the enzyme activity in the following order: DI > BA > CK > DB (**Figure 2**). The BA treatment enhanced UE activity by 190% compared to the CK treatment (P = 0.024), with the enzyme activity in the following order: BA > DI > DB > CK (**Figure 2**).

### 3.5 Soil quality index

**Figure 3:**
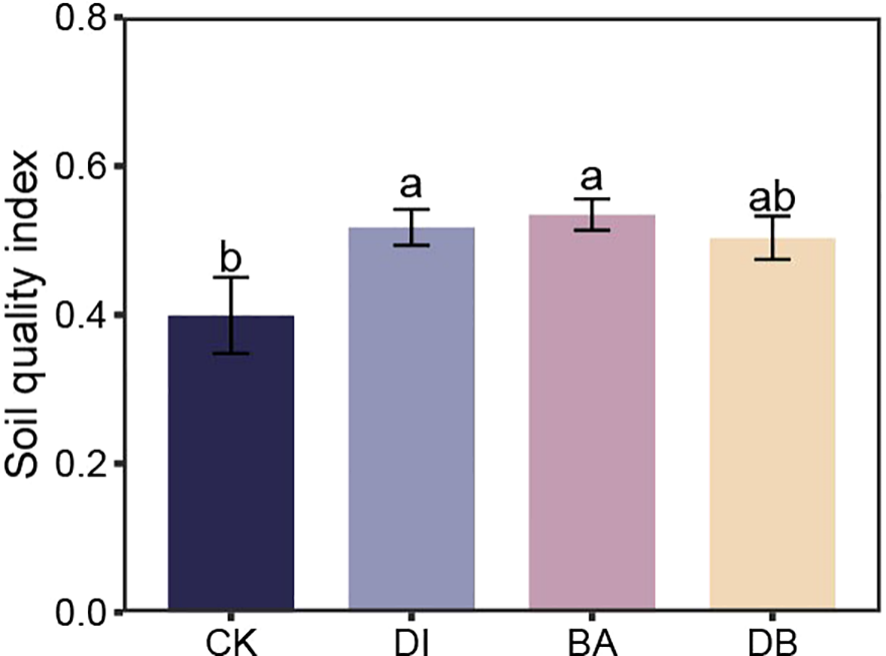
Soil quality index for different treatments. CK, without added crusting agent; DI, added diatom crusting agent; BA, added bacillus crusting agent; DB, added mixed diatom-*B. megaterium* crust. Different letters indicate significant differences at P< 0.05.

PCA identified five principal components (PCs) with eigenvalues ≥ 1, collectively explaining 86.3% of soil attribute variation, which sufficiently retained the original variable information. Based on factor loadings, SC activity (PC1), GMD (PC2), SOC (PC4), and TP (PC5) were selected as the MDS. The calculated SQI values ranged from 0.40 to 0.61. Compared to the CK treatment, DI and BA treatments significantly increased the SQI by 29.7% and 33.9%, respectively (P< 0.05, **Figure 3**).

### 3.6 Ryegrass biomass

**Figure 4:**
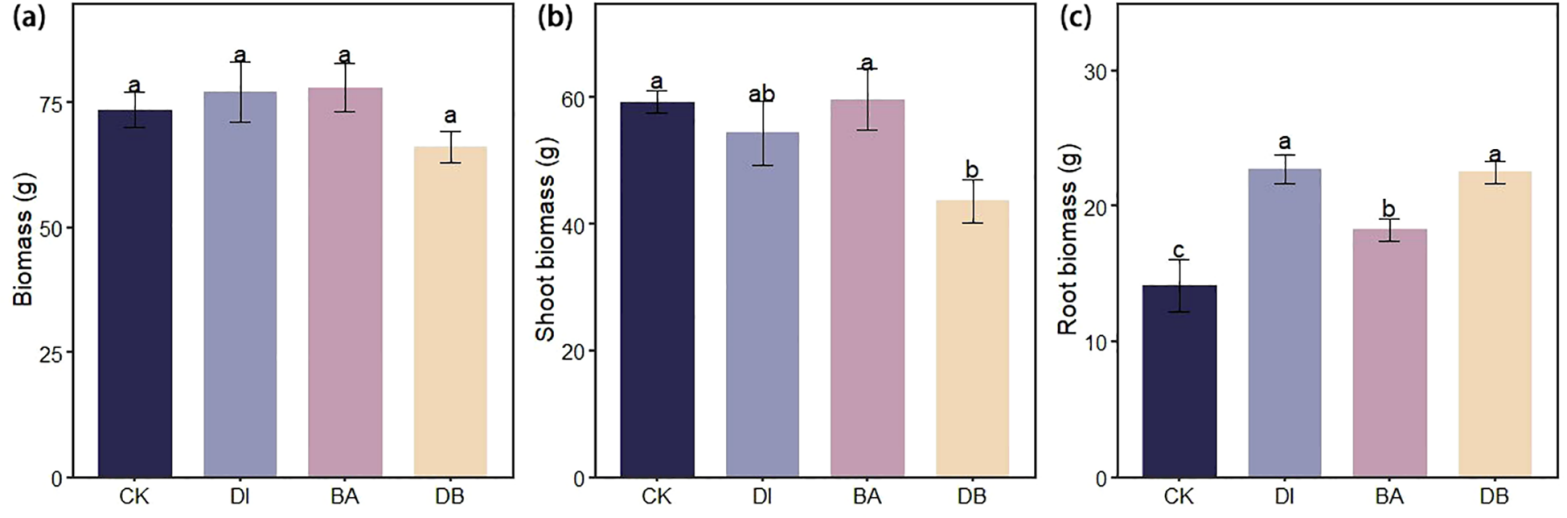
Ryegrass biomass under different biological crust treatments. **(a)** Ryegrass biomass; **(b)** Shoot biomass of ryegrass; And **(c)** Root biomass of ryegrass. CK, without added crusting agent; DI, added diatom crusting agent; BA, added bacillus crusting agent; DB, added mixed diatom-*B. megaterium* crust. Different letters indicate significant differences at P< 0.05.

DI and BA treatments increased total ryegrass biomass by 4.9% and 6.1%, respectively, compared to the CK treatment (**Figure 4A**). Moreover, DI and DB treatments significantly enhanced root biomass by 60.2% and 58.9%, respectively, relative to the CK treatment (**Figure 4C**).

### 3.7 Assessment of the plant–soil system

**Figure 5:**
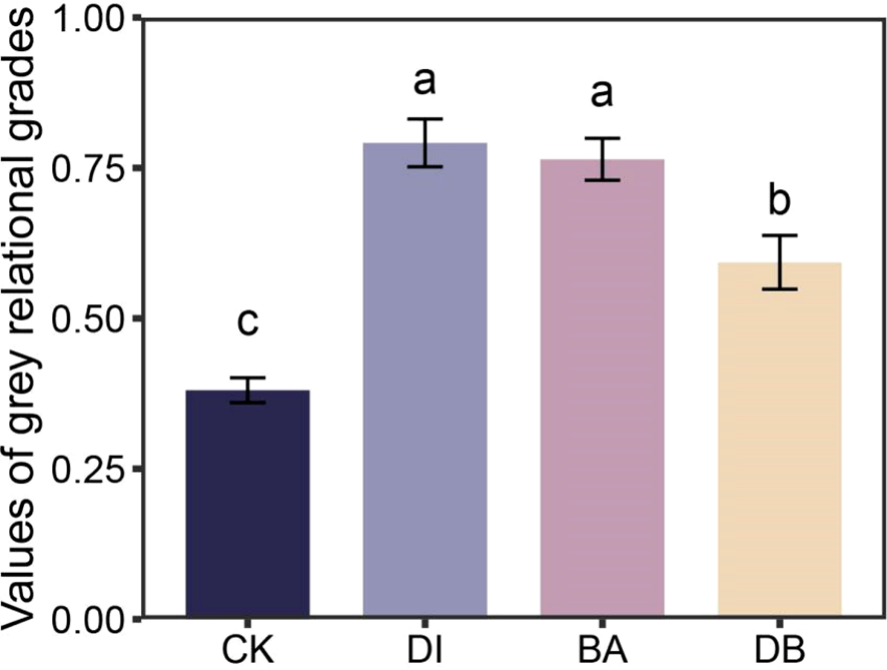
Comparison of grey relational grades of plant–soil system for different treatments. CK, without added crusting agent; DI, added diatom crusting agent; BA, added bacillus crusting agent; DB, added mixed diatom-*B. megaterium* crust. Different letters indicate significant differences at P< 0.05.

The standardization results of the original data and the grey relational coefficients of each index were calculated and are listed in **Supplementary Tables S2**, **S3**. The grey relational grades of the four treatments were subsequently calculated (**Figure 5**). The results showed that the grey values of DI and BA treatments were significantly higher than those of the other two treatments, and the grey relational grade of the DI treatment showed a more favorable trend, reaching a value of 0.79. According to the GRA assessment, the DI treatment is the most suitable treatment for the tested plant-soil system.

### 3.8 Relationships between the SQI and ryegrass root biomass and driving factor analysis of ryegrass root biomass

**Figure 6:**
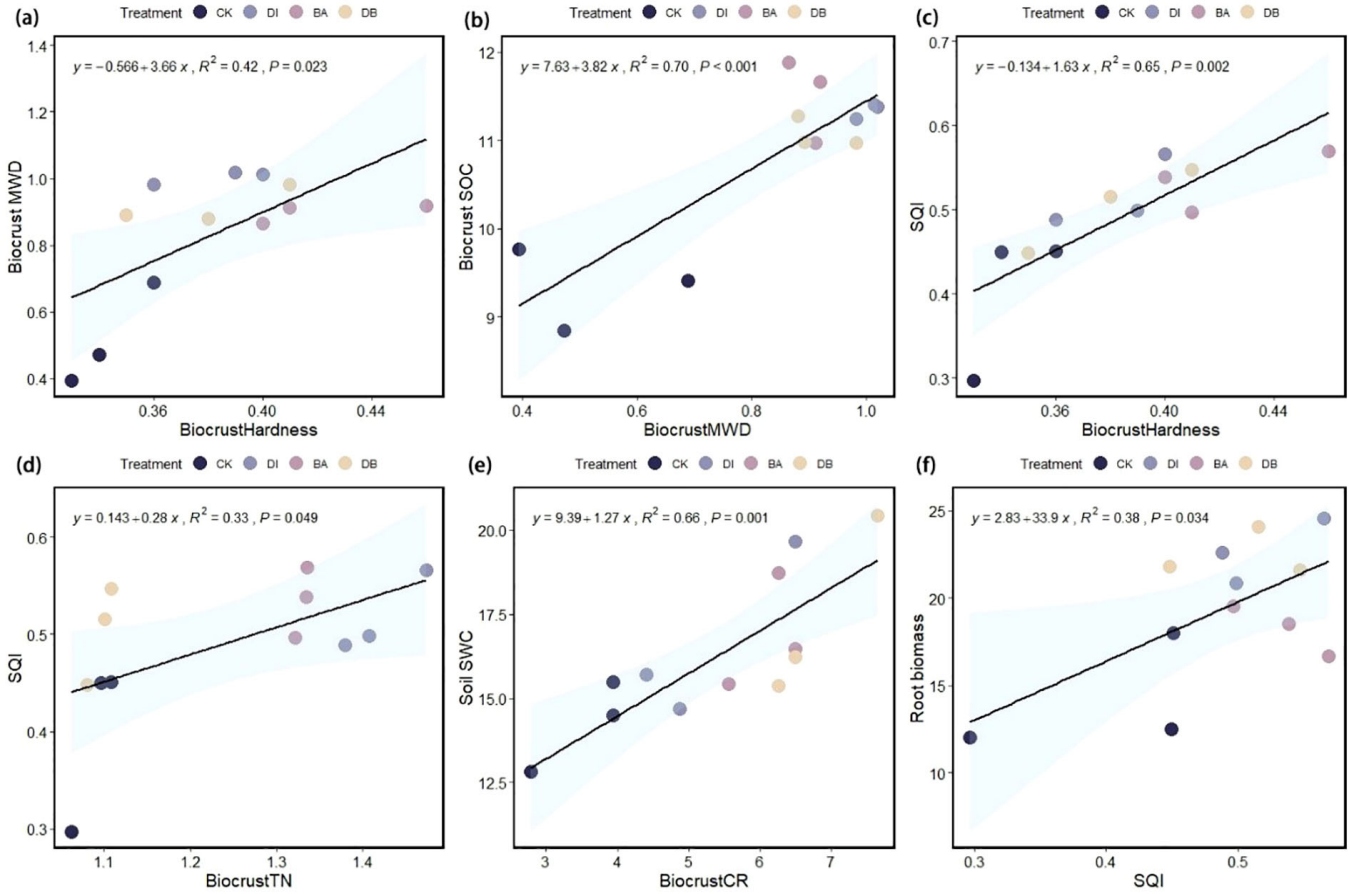
Linear regression analysis. **(a)** Relationship between biocrust hardness and soil MWD; **(b)** relationship between biocrust MWD and biocrust SOC; **(c)** relationship between biocrust hardness and the SQI; **(d)** relationship between biocrust TN content and the SQI; **(e)** relationship between CR and SWC; and **(f)** relationship between the SQI and ryegrass root biomass, mean weight diameter; SOC, soil organic carbon; SQI, Soil Quality Index; TN, total nitrogen; CR, crust roughness; SWC, saturated water content.

**Figure 7:**
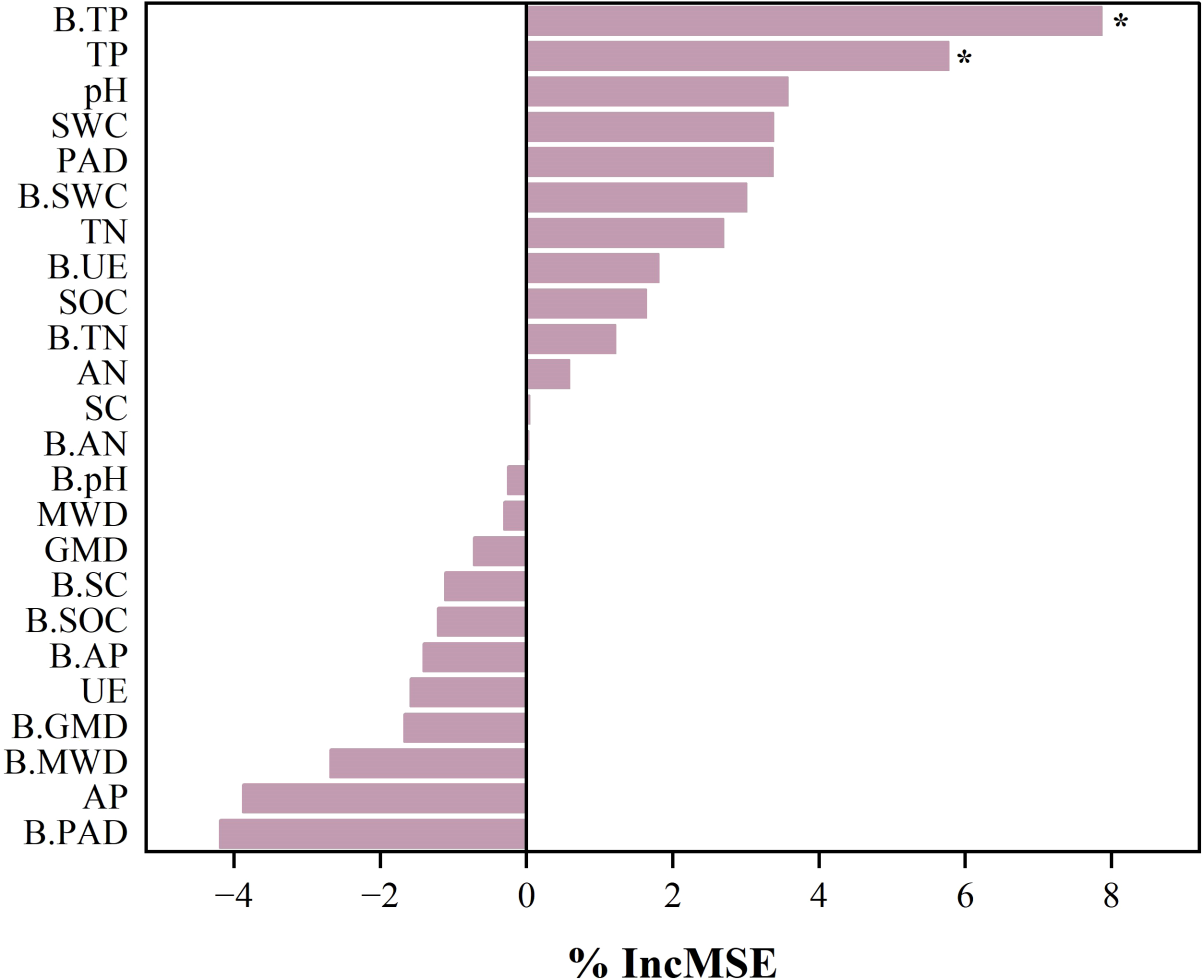
Analysis of drivers affecting root biomass of ryegrass. The random forest method ranks by importance by data. B.SWC, biological soil crusts saturated water content; B.MWD, biological soil crusts mean weight diameter of aggregate; B.GMD, biological soil crusts geometric mean diameter of aggregate; B.PAD, biological soil crusts percentage of aggregate destruction; B.pH, biological soil crusts pH; B.SOC, biological soil crusts organic carbon; B.TN, biological soil crusts total nitrogen; B.TP, biological soil crusts total phosphorus; B.AN, biological soil crusts alkaline nitrogen; B.AP, biological soil crusts available phosphorus; B.SC, biological soil crusts sucrase activity; B.UE, biological soil crusts urease activity; SWC, soil saturated water content; MWD, soil mean weight diameter of aggregate; GMD, soil geometric mean diameter of aggregate; PAD, soil percentage of aggregate destruction; pH; SOC, soil organic carbon; TN, soil total nitrogen; TP, soil total phosphorus; AN, soil alkaline nitrogen; AP, soil available phosphorus; SC, soil sucrase activity; UE, soil urease activity.

The linear regression analysis showed that the SQI explained 38% of the variance in root biomass (P< 0.05, **Figure 6F**), indicating its significance as a root growth promoter. Crust hardness and TN content explained 65% and 33% of SQI variance, respectively (P< 0.05), identifying them as the key indicators influencing the SQI (**Figures 6C, D**). Additionally, crust hardness was a critical factor affecting the MWD (**Figure 6A**), while CR significantly influenced the SWC (**Figure 6E**). Random forest analysis further revealed the key factors affecting the subsurface biomass of ryegrass (**Figure 7**), specifically, biocrust TP and soil TP were identified as significant positive predictors of root biomass of ryegrass (P< 0.01).

## 4 Discussion

### 4.1 Effect through which different types of biocrusts improved the SQI in the mining subsidence area

Our results indicate that the sole application of DI biocrust enhances the SQI value (**Figure 3**). Research results show that DI-treated crusts exhibited elevated surface hardness (**Table 1**). DI crusts bind soil particles through the secretion of extracellular polysaccharides (EPS), forming a weakly cemented layer that increases structural integrity (Belnap, 2006). These consolidated surfaces preserve soil structural stability against external stress factors (Pointing and Belnap, 2012), as evidenced by improved aggregate cohesion in algal crusts (**Table 1**). Enhanced aggregation facilitates organic matter retention (**Figure 6B**), thereby supporting soil quality improvement (Elliott, 1986). The crust TN content also showed a strong correlation with the SQI (**Figure 6D**), implying that TN is a critical quality determinant. DI crusts displayed a higher TN content (**Figure 1**), attributable to EPS-facilitated capture of aeolian nutrient deposits within the cementation layer (Rillig et al., 2014). Similarly, the application of the exclusive bacterial crust (BA) elevated the SQI (**Figure 3**). Bacterial networks and hydrophobic proteins facilitate the formation of a high-hardness crust (Tisdale et al., 1985), consistent with the measured surface rigidity of the BA crust (**Table 2**). The linear regression analysis revealed a significant positive correlation between crust hardness and the SQI (**Figure 6D**). This relationship may stem from the metabolic activity of *B. megaterium*. Bacterial pyruvate conversion yields organic acids (e.g., lactic acid and acetic acid) (Goswami et al., 2018), which acidify the soil to stimulate enzymatic activity. Consequently, the nitrogen/phosphorus transformation process is accelerated, releasing organically bound nutrients (Wu et al., 2024). This mechanism is supported by the observation that BA-treated crusts exhibited superior UE activity (**Figure 1**), which directly regulates nitrogen mineralization and bioavailability. Conversely, mixed diatom-*B. megaterium* crust (DB) did not improve the SQI (**Figure 3**). Nutrient competition (particularly nitrogen/phosphorus) between microorganisms likely induced net nutrient depletion (Maestre et al., 2011), which is reflected by the minimal soil TN content in the DB-treated crust (**Figure 3**). Additionally, bacterial dominance in phosphate utilization may suppress algal growth (Kirchman, 1994), further impeding nutrient accrual in co-inoculated systems.

### 4.2 Effects of treatment with different biocrusts on ryegrass root biomass and its influencing factors

**Figure 8:**
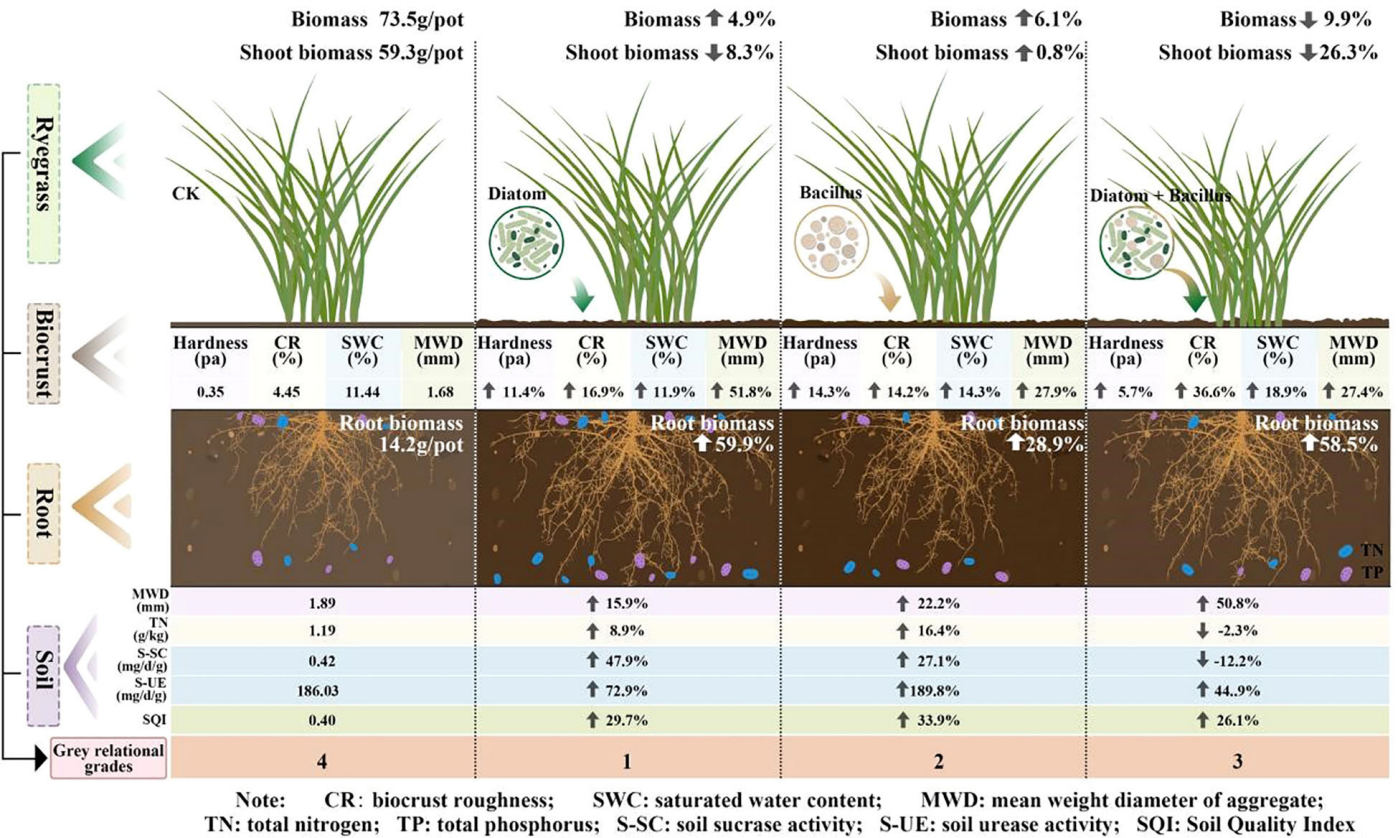
Graphical abstract.

The linear regression analysis indicated a positive correlation between plant root biomass and SQI (**Figure 6F**). The DI treatment significantly enhanced ryegrass root biomass (**Figure 4C**) and achieved optimal plant-soil system integration based on the grey correlation analysis (**Figure 5**). Mechanistically, DI-treated crusts exhibited moderate surface roughness (**Table 2**), which provides microsites for seed retention and moisture conservation; this finding is consistent with the observed crust and soil moisture patterns (**Tables 1**, **2**). This textural feature is derived from EPS-mediated crust formation, where desiccation induces cracking that amplifies surface heterogeneity (Chamizo et al., 2012). Additionally, EPS promotes soil particle aggregation (Rodríguez-Caballero et al., 2018; Roman et al., 2021), improving soil structure to enhance aeration and microecological conditions. This structural optimization facilitates root development, nutrient acquisition, and metabolic activity (Wang et al., 2015). Phosphorus in the crust and soil is a key driver of root biomass of ryegrass (**Figure 7**), we hypothesize that the pH of algal crust decreases due to CO_2_ consumption through its own photosynthesis (**Figure 1**), and the soil pH is closer to the optimal dissolution range of phosphorus 6-7, which accelerates the dissolution of phosphorus and increases the content of available phosphorus in soil (**Figure 1**) (Zhu et al., 2018). Similarly, the DB treatment increased ryegrass root biomass (**Figure 4C**). The enhanced surface roughness of the DB crust (**Table 1**) probably results from synergistic interactions: photosynthetic diatoms provide carbohydrates and bacterial hyphae create structural frameworks (Mallen-Cooper et al., 2019). This partnership improves soil aggregation efficiency through combined hyphal-EPS binding (Delgado-Baquerizo et al., 2016; Fan et al., 2020), thereby stabilizing surface structure to support root penetration. Simultaneously, photoautotrophic diatoms support heterotrophic bacterial metabolism by supplying respiratory substrates that stimulate hyphal production (Rossi et al., 2018), enhancing microbial activity and nutrient cycling to benefit root development. However, the suboptimal grey correlation score of the DB crust suggests potential limitations for sustained plant growth. The increase in root biomass under DB treatment was mainly due to the improved microtopography and water retention capacity of the crust roughness (**Table 1**), which provided a better physical space for root growth. Nutrients may also be fixed in their biomass by microorganisms, which contributes to the increase of root biomass under DB treatment, but reduces the available nutrients in the soil and does not increase SQI (**Figure 8**). At the same time, in soils with reduced nutrients, plants focus on forming well-developed root systems to cope with nutrient stress (Hermans et al., 2006). However, the rapid expansion of the root system in the early stages, without a continuous nutrient supply, may lead to more severe nutrient stress in the later stages of growth, limiting its long-term survival and growth (**Figure 4B**). However, if soil nutrients are supplemented in later stages, it may benefit plant growth (Freschet et al., 2021). BA treated soils have lower AP levels (**Figure 2**), which we speculate is due to the fact that bacterial lichens can dissolve mineral phosphorus (Baumann et al., 2019; Qi et al., 2021), However, in nutrient-poor soils, Bacillus colonies compete with plants for limited phosphorus resources (Harper and Jayne, 2001), limiting root development. Nutrient data from plant tissues can provide sufficient evidence to support the “nutrient competition” hypothesis. However, due to the limitation of the experimental period, this study failed to measure these data and used plant biomass as an indicator to support plant growth.

## 5 Conclusion

In the present study, the *Bacillus*-dominated crust enhanced the SQI of the soil; however, it failed to significantly promote ryegrass root biomass. In contrast, the diatom-based biocrust exhibited a more favorable performance by improving soil structure; increasing the contents of SOC, TN, and AP in the soil surface layer, and significantly enhancing ryegrass root biomass. The mixed diatom-*B. megaterium* crust enhanced soil aggregate stability and microbial activity by forming a crust layer with high surface roughness, thereby facilitating the increase in ryegrass root biomass. Nevertheless, the interspecific competition for nitrogen and phosphorus resources between the two components of the mixed crust hindered SQI improvement. The single diatom crust treatment effectively improved soil structure and nutrient content in the mining subsidence area and promoted plant root growth, providing a theoretical foundation for further research on using biocrusts to remediate such areas. Because this study used a pot experiment design with controlled conditions, it has inherent limitations. Future research should prioritize the determination of the optimal dose and application methods of biocrusts. Field trials are also essential to validate the effects of diatom-based crusting agents on the SQI and plant growth under natural environmental conditions, bridging the gap between controlled experiments and real-world applications.

## Data Availability

The raw data supporting the conclusions of this article will be made available by the authors, without undue reservation.
