# Supplementary material for: Bacillus megaterium and diatom improve mineral mining area soil quality and root biomass individually but show slightly inferior combined effects
Source: Front Plant Sci. 2025 Dec 16;16:1716914. doi: 10.3389/fpls.2025.1716914 (PMC12748183; doi:10.3389/fpls.2025.1716914)
Supplement: Supplementary file 1 [file DataSheet1.docx]

**Table S1.**

PCA loadings for all factors

| Treatments | SC | AN | PAD | pH | TN | UE | GMD | MWD | SOC | TP | AP | SWC |
| --- | --- | --- | --- | --- | --- | --- | --- | --- | --- | --- | --- | --- |
| PC1 | **0.91** | **0.75** | **-0.69** | **-0.68** | **0.61** | **0.54** | -0.08 | -0.22 | -0.02 | 0.05 | 0.38 | 0.25 |
| PC2 | -0.19 | -0.02 | 0.24 | 0.07 | 0.14 | 0.47 | **0.91** | **0.88** | 0.07 | 0.32 | 0.49 | 0.52 |
| PC3 | -0.12 | -0.5 | -0.07 | 0.46 | 0.58 | 0.53 | -0.31 | -0.34 | 0.46 | 0.22 | -0.06 | 0.25 |
| PC4 | 0.03 | 0.16 | 0.57 | -0.28 | -0.19 | 0.31 | -0.04 | -0.09 | **0.75** | -0.56 | 0.20 | -0.17 |
| PC5 | 0.08 | 0.07 | 0.21 | -0.32 | 0.33 | -0.06 | 0.19 | 0.16 | 0.30 | **0.60** | **-0.54** | **-0.54** |

**Table S2.**

Processing results for the standardization of the original data

| Treatments | CK | DI | BA | DB |
| --- | --- | --- | --- | --- |
| Biocrust hardness (X_1_) | 0 | 0.75 | 1 | 0.5 |
| CR (X_2_) | 0 | 0.46 | 0.39 | 1 |
| Biocrust SWC (X_3_) | 0 | 0.63 | 0.76 | 1 |
| Biocrust MWD (X_4_) | 0 | 1 | 0.54 | 0.53 |
| Biocrust GMD (X_5_) | 0 | 1 | 0.57 | 0.49 |
| Biocrust water-stable MWD (X_6_) | 0 | 1 | 0.78 | 0.82 |
| Biocrust water-stable GMD (X_7_) | 0 | 1 | 0.81 | 0.89 |
| Biocrust PAD (X_8_) | 0 | 1 | 0.83 | 0.99 |
| Biocrust pH (X_9_) | 0 | 1 | 0.82 | 0.26 |
| Biocrust SOC (X_10_) | 0 | 0.92 | 1 | 0.80 |
| Biocrust TN (X_11_) | 0 | 1 | 0.73 | 0.02 |
| Biocrust TP (X_12_) | 0.01 | 0.33 | 1 | 0 |
| Biocrust AN (X_13_) | 0.22 | 1 | 0.78 | 0 |
| Biocrust AP (X_14_) | 0 | 1 | 0.78 | 0.47 |
| Biocrust SC (X_15_) | 0.42 | 0.36 | 1 | 0 |
| Biocrust UE (X_16_) | 0 | 0.82 | 1 | 0.25 |
| SWC (X_17_) | 0.33 | 0.70 | 0.76 | 1 |
| MWD (X_18_) | 0.33 | 0.42 | 0.47 | 1 |
| GMD (X_19_) | 0.33 | 0.46 | 0.58 | 1 |
| Water-stable MWD (X_20_) | 0 | 0.68 | 1 | 0.69 |
| Water-stable GMD  (X_21_) | 0 | 1 | 0.72 | 0.60 |
| PAD (X_22_) | 0.26 | 1 | 0.45 | 0 |
| pH (X_23_) | 0.18 | 1 | 0.82 | 0 |
| SOC (X_24_) | 0.73 | 0 | 1 | 0.46 |
| TN (X_25_) | 0.12 | 0.61 | 1 | 0 |
| TP (X_26_) | 0 | 0.92 | 0.99 | 1 |
| AN (X_27_) | 0.22 | 1 | 0.78 | 0 |
| AP (X_28_) | 0 | 1 | 0.38 | 0.75 |
| SC (X_29_) | 0.22 | 1 | 0.65 | 0 |
| UE (X_30_) | 0 | 0.78 | 0.85 | 1 |
| SQI (X_31_) | 0 | 0.87 | 1 | 0.77 |
| Biomass (X_32_) | 0.62 | 0.92 | 1 | 0 |
| Shoot biomass (X_33_) | 0.97 | 0.67 | 1 | 0 |
| Root biomass (X_34_) | 0 | 1 | 0.48 | 0.98 |

**Table S3.**

Calculated values of the grey relational coefficient η_i_(k)

| Treatments | CK | DI | BA | DB |
| --- | --- | --- | --- | --- |
| X_1_ | 0 | 0.75 | 1 | 0.5 |
| X_2_ | 0 | 0.46 | 0.39 | 1 |
| X_3_ | 0 | 0.63 | 0.76 | 1 |
| X_4_ | 0 | 1 | 0.54 | 0.53 |
| X_5_ | 0.33 | 1 | 0.54 | 0.49 |
| X_6_ | 0.33 | 1 | 0.70 | 0.74 |
| X_7_ | 0.33 | 1 | 0.72 | 0.82 |
| X_8_ | 0 | 1 | 0.75 | 0.99 |
| X_9_ | 0.33 | 1 | 0.73 | 0.40 |
| X_10_ | 0.33 | 0.87 | 1 | 0.71 |
| X_11_ | 0.33 | 1 | 0.65 | 0.34 |
| X_12_ | 0.33 | 0.43 | 1 | 0.33 |
| X_13_ | 0.39 | 1 | 0.69 | 0.33 |
| X_14_ | 0.33 | 1 | 0.70 | 0.48 |
| X_15_ | 0.46 | 0.44 | 1 | 0.33 |
| X_16_ | 0.33 | 0.74 | 1 | 0.40 |
| X_17_ | 0.33 | 0.70 | 0.76 | 1 |
| X_18_ | 0.33 | 0.42 | 0.47 | 1 |
| X_19_ | 0.33 | 0.46 | 0.58 | 1 |
| X_20_ | 0 | 1 | 0.68 | 0.69 |
| X_21_ | 0.33 | 1 | 0.64 | 0.56 |
| X_22_ | 0.40 | 1 | 0.47 | 0.33 |
| X_23_ | 0.38 | 1 | 0.73 | 0.33 |
| X_24_ | 0.65 | 0.33 | 1 | 0.48 |
| X_25_ | 0.36 | 0.56 | 1 | 0.33 |
| X_26_ | 0.33 | 0.87 | 0.99 | 1 |
| X_27_ | 0.39 | 1 | 0.69 | 0.33 |
| X_28_ | 0.33 | 1 | 0.45 | 0.67 |
| X_29_ | 0.39 | 1 | 0.59 | 0.33 |
| X_30_ | 0 | 0.78 | 0.85 | 1 |
| X_31_ | 0.33 | 0.45 | 1 | 0.40 |
| X_32_ | 0.57 | 0.87 | 1 | 0.33 |
| X_33_ | 0.95 | 0.6 | 1 | 0.33 |
| X_34_ | 0.33 | 1 | 0.49 | 0.96 |
